# The Role of the Renin–Angiotensin–Aldosterone System in Hypertensive Emergencies

**DOI:** 10.3390/biomedicines14091899

**Published:** 2026-08-26

**Authors:** Tatiana Palotta Minari, Luciana Neves Cosenso-Martin, Jessica Rodrigues Roma Uyemura, Aleandra Marton Polegati Santos, Valquíria da Silva Lopes, Rauer Ferreira Franco, Marco Antônio Vieira-da-Silva, Kléber Aparecido de Oliveira, Marco Aurélio de Almeida, Juan Carlos Yugar-Toledo, José Fernando Vilela-Martin

**Affiliations:** 1 Base Hospital, FUNFARME, São José do Rio Preto 15090-000, SP, Brazil; luciana-martin@uol.com.br (L.N.C.-M.); jessicauyemura@gmail.com (J.R.R.U.); aleandrapolegati@hotmail.com (A.M.P.S.); valquiria_lopes_2009@hotmail.com (V.d.S.L.); rauer.franco@edu.famerp.br (R.F.F.); marcovieiravs@gmail.com (M.A.V.-d.-S.); kleberapoliveira095@gmail.com (K.A.d.O.); marco.almeida@uftm.edu.br (M.A.d.A.); yugarjuan@uol.com.br (J.C.Y.-T.); vilelamartin@uol.com.br (J.F.V.-M.); 2 Medical School São José do Rio Preto—FAMERP, São José do Rio Preto 15090-000, SP, Brazil

**Keywords:** renin–angiotensin–aldosterone system, hypertensive emergency, angiotensin II, aldosterone, endothelial dysfunction, oxidative stress, inflammation, vascular injury, target organ damage, cardiovascular disease

## Abstract

**Background:** Hypertensive emergencies are severe clinical conditions characterized by sudden elevation of blood pressure accompanied by acute target organ damage, including cardiovascular, cerebrovascular, renal, and retinal complications. Although blood pressure elevation is the defining feature, accumulating evidence indicates that activation of the renin–angiotensin–aldosterone system (RAAS) may play a central role in the molecular and cellular mechanisms underlying vascular injury. This review critically examines the current evidence regarding the involvement of the RAAS in the pathophysiology of hypertensive emergencies, highlighting its contribution to endothelial dysfunction, oxidative stress, inflammation, microvascular damage, and target organ injury, as well as its therapeutic implications and future research perspectives. **Methods:** A comprehensive narrative review of the literature was conducted using narrative reviews, systematic reviews, meta-analyses, experimental, translational, and clinical studies published in major biomedical databases, with particular emphasis on recent evidence addressing RAAS signaling, hypertensive emergencies, target organ damage, and emerging therapeutic approaches. Current international guidelines and landmark studies were also considered to provide an updated overview of the diseases. **Results:** Available evidence indicates that excessive activation of the classical ACE/angiotensin II/AT1 receptor axis contributes to acute vasoconstriction, endothelial dysfunction, reactive oxygen species generation, inflammatory activation, impaired vascular autoregulation, and microvascular injury, thereby promoting acute damage to the brain, heart, kidneys, retina, and large arteries. Conversely, the counter-regulatory ACE2/angiotensin-(1–7)/Mas receptor axis exerts protective vascular effects, although its role in hypertensive emergencies is unclear. Current therapies primarily focus on controlled blood pressure reduction, whereas the potential benefits of targeted RAAS modulation during the acute phase remain uncertain. **Conclusions:** The RAAS may play an important role in the pathophysiological processes underlying hypertensive emergencies, extending beyond its classical function of blood pressure regulation. However, direct human evidence remains limited and heterogeneous, warranting caution in interpreting these findings. A deeper understanding of the interactions between RAAS activation, vascular dysfunction, and target organ injury could help identify potential biomarkers and therapeutic targets, ultimately contributing to improved management and prognosis in patients with hypertensive emergencies.

## 1. Introduction

Hypertension remains the leading modifiable risk factor for cardiovascular morbidity and mortality worldwide, affecting more than one billion people and accounting for approximately 10 million deaths annually [1]. Although most patients with hypertension experience a chronic and relatively stable clinical course, a small but clinically significant proportion develop hypertensive emergencies, characterized by an abrupt elevation in blood pressure associated with acute target organ injury involving the brain, heart, kidneys, retina, or large arteries [2,3]. These conditions require immediate recognition and prompt blood pressure reduction, as delayed treatment substantially increases the risk of irreversible organ dysfunction and death [4].

The pathophysiology of hypertensive emergencies is considerably more complex than a simple acute increase in systemic arterial pressure [5]. Rather than representing only a hemodynamic disorder, these emergencies involve a cascade of molecular, cellular, and vascular events that amplify tissue injury [6]. Excessive mechanical stress on the vascular wall disrupts endothelial integrity, impairs autoregulation, increases permeability, and initiates inflammatory and thrombotic responses [7]. This vicious cycle promotes ischemia and accelerates target organ damage, even when blood pressure elevation is relatively short-lived [5,6,7].

Among the implicated neurohormonal systems, the renin–angiotensin–aldosterone system (RAAS) has been proposed as a relevant contributor linking acute hypertension to vascular and organ injuries [8,9]. Beyond its classical role in sodium balance and systemic pressure regulation [10], RAAS activation, particularly via angiotensin II, has been associated with vasoconstriction, oxidative stress, inflammatory cytokine release, endothelial dysfunction, sympathetic activation, and vascular remodeling [9,10,11]. Evidence from hypertensive emergencies suggests that local RAAS activation within the heart, kidneys, cerebral circulation, and vascular wall may exacerbate acute target organ injury [12,13,14]. Conversely, the counter-regulatory ACE2/Ang-(1–7)/Mas axis exerts vasodilatory and anti-inflammatory effects, and an imbalance between these pathways has been implicated in acute syndromes characterized by endothelial injury and inflammation [15,16,17].

Nevertheless, the precise contribution of the RAAS to hypertensive emergencies remains unclear [15,18,19,20]. Clinical studies assessing circulating renin, aldosterone, and angiotensin peptides have produced heterogeneous results, influenced by disease heterogeneity, timing of sampling, prior therapy, and biological variability [21,22]. While RAAS inhibitors are central to chronic hypertension management, their role in acute hypertensive emergencies is limited, with current treatment relying mainly on titratable intravenous vasodilators and beta-blockers [16,23,24]. Whether selective modulation of RAAS pathways can improve outcomes beyond blood pressure reduction remains unclear [15,16,17,25].

Given these uncertainties, a cautious reassessment of the role of RAAS in hypertensive emergencies is warranted, emphasizing the need for further studies to clarify its mechanistic and therapeutic relevance. This narrative review critically examines current experimental and clinical evidence regarding the involvement of the RAAS in the pathogenesis of hypertensive emergencies, with particular emphasis on the molecular mechanisms, endothelial dysfunction, oxidative stress, inflammation, microvascular injury, and target organ damage associated with this condition. In addition, this article discusses the therapeutic implications of RAAS modulation, identifies current knowledge gaps, and highlights emerging research directions that may contribute to the development of more precise diagnostic and therapeutic strategies for this severe cardiovascular condition.

## 2. Methods

This narrative review provides a comprehensive and critical overview of the current evidence regarding the role of the RAAS in hypertensive emergencies. A structured literature search was performed in PubMed/MEDLINE, Scopus, and Web of Science on 15 July 2026. The search strategy combined controlled vocabulary (Medical Subject Headings–MeSH) and free-text terms related to hypertensive emergencies and the RAAS. The Boolean operators used were AND and OR. For example, the complete PubMed search string was as follows: (“hypertensive emergency” OR “hypertensive crisis” OR “malignant hypertension”) AND (“renin-angiotensin system” OR “renin-angiotensin-aldosterone system” OR “angiotensin II” OR “aldosterone” OR “ACE” OR “ACE2” OR “AT1 receptor” OR “Mas receptor”) AND (“endothelial dysfunction” OR “oxidative stress” OR “vascular injury” OR “microvascular dysfunction” OR “inflammation” OR “target organ damage” OR “vascular remodeling”). Equivalent strategies were adapted for Scopus and the Web of Science to ensure consistency across databases.

The search identified 165 records in total. After the removal of duplicates, titles and abstracts were screened, resulting in 110 articles being selected for the full-text review. Of these, 90 met the predefined inclusion criteria and were included in the analysis. Studies were included if they were original experimental, translational, or clinical investigations addressing RAAS mechanisms in hypertensive emergencies, as well as systematic reviews, meta-analyses, narrative reviews with mechanistic insights, clinical guidelines, and landmark experimental investigations published in peer-reviewed journals. Only articles published in English were considered. Studies were excluded if they focused exclusively on chronic hypertension without relevance to acute hypertensive target organ injury, or if they were case reports, conference abstracts, or non-peer-reviewed publications.

Study selection was performed independently by two reviewers, and disagreements were resolved by consensus. To evaluate the strength of the evidence, studies were qualitatively appraised according to their design, sample size, methodological rigor, and relevance to hypertensive emergencies. Although a formal risk-of-bias tool was not applied, the evidence was classified as high when derived from systematic reviews, meta-analyses, or large clinical trials, moderate when based on smaller clinical studies or translational investigations, and low when limited to experimental studies with uncertain applicability to humans.

The final body of evidence was organized into thematic sections addressing the physiological organization of the RAAS, molecular and cellular mechanisms implicated in hypertensive emergencies, organ-specific manifestations, therapeutic implications, emerging concepts, knowledge gaps, and future research directions.

## 3. Review

### 3.1. Hypertensive Emergencies: Definition and Current Concepts

Hypertensive emergencies are severe clinical syndromes characterized by sudden and marked elevation in blood pressure, accompanied by acute or ongoing target organ damage that requires immediate blood pressure reduction to prevent irreversible injury and death [1]. Although no absolute blood pressure threshold defines a hypertensive emergency, systolic blood pressure exceeding 180 mmHg and/or diastolic blood pressure exceeding 120 mmHg are commonly observed [2]. Importantly, the diagnosis is established by the presence of acute target organ injury rather than by blood pressure values alone, as some individuals tolerate substantially higher blood pressure levels without developing acute complications, whereas others may experience severe organ damage at comparatively lower pressures, depending on baseline vascular adaptation, comorbidities, and rate of blood pressure elevation [1,3].

Hypertensive emergencies account for approximately 1–2% of emergency department visits related to hypertension but are associated with considerable morbidity, prolonged hospitalization, and high short- and long-term mortality rates [4]. Despite advances in antihypertensive therapy and improved awareness of hypertension, the incidence of hypertensive emergencies has not declined substantially in many countries, largely because of poor blood pressure control, population aging, increasing obesity prevalence, chronic kidney disease, and nonadherence to medication [5]. Patients with resistant hypertension, secondary hypertension, chronic kidney disease, diabetes mellitus, and previous cardiovascular disease are particularly susceptible to developing acute hypertensive complications [5,6,7].

The clinical presentation of hypertensive emergencies is highly heterogeneous and depends primarily on the affected organ system [8,9]. Neurological manifestations include hypertensive encephalopathy, ischemic stroke, intracerebral hemorrhage, posterior reversible encephalopathy syndrome (PRES), seizures, and altered consciousness [10]. Cardiovascular complications include acute heart failure with pulmonary edema, acute coronary syndrome, myocardial ischemia, and acute aortic syndrome. Renal involvement ranges from acute kidney injury and rapidly progressive renal dysfunction to thrombotic microangiopathy and severe proteinuria [2,11]. Ocular manifestations, including retinal hemorrhage, cotton-wool spots, hard exudates, and papilledema, reflect advanced retinal microvascular injury and frequently coexist with systemic vascular injuries. These clinical phenotypes often overlap, emphasizing the systemic nature of hypertensive emergencies [9,10,11]. Clinical–epidemiological studies have shown that hypertensive emergencies occur predominantly in older patients, often sedentary, aware of hypertension, and with lower rates of antihypertensive treatment [12]. Clinical and biochemical–metabolic profiles are altered in hypertensive emergencies and are linked to hyperglycemia, dyslipidemia (lower HDL cholesterol), higher creatinine levels with reduced kidney function, and lower serum potassium levels [13].

Historically, hypertensive emergencies have been attributed mainly to excessive intravascular pressure, which overwhelms vascular autoregulation [14,15]. However, current evidence shows that they represent complex multisystem disorders involving endothelial dysfunction, neurohormonal activation, inflammation, oxidative stress, coagulation abnormalities, and microvascular injury [16]. Abrupt blood pressure elevation damages endothelial cells, disrupts the glycocalyx, increases permeability, and activates platelets and leukocytes, initiating a cycle of vascular injury that perpetuates ischemia, independently of systemic pressure [15,16,17,18]. The failure of autoregulation in the cerebral, coronary, and renal circulations further contributes to capillary leakage, fibrinoid necrosis, and microvascular thrombosis, explaining why ischemic injury often accompanies severe hypertension [2,19,20,21,22,23].

Among the neurohormonal systems involved, the RAAS has been implicated as a contributor to acute vascular and organ injuries [15,23,24,25,26]. Activation of the ACE/Ang II/AT1 receptor axis promotes vasoconstriction, oxidative stress, inflammatory signaling, and endothelial dysfunction, which occur rapidly and independently of blood pressure [17]. Studies have demonstrated that Ang II stimulates NADPH oxidases, increases reactive oxygen species, reduces nitric oxide bioavailability, and activates NF-κB, leading to acute endothelial dysfunction and microvascular damage [16]. In contrast, the ACE2/Ang-(1–7)/Mas receptor axis exerts vasodilatory, antioxidant, and anti-inflammatory effects; however, during hypertensive emergencies, this pathway appears insufficient to counterbalance excessive Ang II signaling [17]. While this imbalance is well established during chronic hypertension, its dynamics in acute crises remain poorly defined [15,16,17].

The discovery of ACE2 revealed a counter-regulatory pathway within the RAAS that metabolizes Ang II into Ang-(1–7), thereby reducing Ang II availability and generating a biologically protective peptide [17,27,28,29,30,31,32]. Acting through the Mas receptor, Ang-(1–7) stimulates nitric oxide production, suppresses oxidative stress, inhibits inflammatory signaling, and promotes vasodilation, mechanisms that under physiological conditions contribute to vascular protection [15,16,17]. Studies suggest that enhancement of this pathway can attenuate hypertension-induced vascular injury [17], whereas reduced ACE2 activity amplifies AT1R-mediated damage [15,16,17]. However, despite its recognized role in hypertension and other cardiovascular syndromes [33,34,35,36], the contribution of the ACE2/Ang-(1–7)/Mas receptor axis in hypertensive emergencies remains unclear. Preliminary evidence indicates that acute endothelial injury and oxidative stress may reduce ACE2 activity, shifting the RAAS toward excessive Ang II signaling [17,37,38]; however, whether restoration of ACE2 function represents a feasible therapeutic strategy in the acute setting is uncertain and requires further investigation [15,16,17,39,40,41,42,43,44,45].

### 3.2. Tissue-Specific RAAS

One of the most important advances in cardiovascular biology is the recognition that the RAAS functions not only as a circulating endocrine system but also as a collection of tissue-specific regulatory networks. Local synthesis of renin, angiotensin peptides, ACE, ACE2, and their receptors has been demonstrated in multiple organs, enabling the autonomous regulation of cellular function independent of systemic hormone concentrations [46,47,48].

Within the vascular wall, locally produced Ang II acutely modulates vascular tone, endothelial permeability, and inflammatory responses, thereby amplifying endothelial dysfunction and microvascular injury during hypertensive emergencies. While chronic consequences such as arterial remodeling, stiffness, and rarefaction are well established, in the acute setting, vascular RAAS activation is particularly relevant for ischemic injury [49,50,51,52].

Cardiac RAAS activation contributes to oxidative stress, contractile dysfunction, and instability. These mechanisms, beyond their role in chronic hypertrophy and fibrosis, may precipitate acute myocardial ischemia or pulmonary edema in hypertensive emergencies [53,54].

Renal RAAS activation is particularly significant because the kidney is both a regulator and target of this system. Intrarenal Ang II concentrations often exceed circulating levels because of local synthesis and intracellular accumulation [2,15,16,17,49]. Excessive activation induces arteriolar vasoconstriction, glomerular hypertension, and tubular inflammation, mechanisms that exacerbate acute kidney injury and accelerate renal deterioration during hypertensive crises [55,56].

Similarly, the cerebral RAAS plays a critical role in regulating cerebral blood flow, blood–brain barrier integrity, and autonomic control of the cardiovascular system. Experimental models have shown that excessive brain Ang II signaling promotes oxidative stress, endothelial dysfunction, barrier disruption, and microglial activation, changes that may contribute to hypertensive encephalopathy, posterior reversible encephalopathy syndrome, intracerebral hemorrhage, and ischemic stroke [15,16,17,57,58,59,60].

### 3.3. RAAS as an Integrative Network in Hypertensive Emergencies

Rather than acting as an isolated hormonal cascade, the RAAS functions as an integrative signaling network that interacts extensively with the sympathetic nervous system, endothelin signaling, nitric oxide metabolism, oxidative stress pathways, inflammatory mediators, coagulation cascades, and innate and adaptive immune responses [61]. During hypertensive emergencies, these interconnected pathways amplify each other, producing a self-perpetuating cycle of endothelial injury, impaired autoregulation, vasoconstriction, microvascular thrombosis, and progressive organ damage [62,63,64]. Therefore, the current understanding of RAAS biology extends far beyond its traditional role in blood pressure regulation. The balance between the deleterious ACE/Ang II/AT1R pathway and the protective ACE2/Ang-(1–7)/Mas receptor axis appears to determine the magnitude of vascular injury during acute hypertensive states [15,16,17]. Moreover, the growing recognition of tissue-specific RAAS activation provides a mechanistic framework for explaining why target organ damage may continue to progress even after systemic blood pressure is reduced [65,66,67,68]. These concepts form the basis for understanding the molecular mechanisms discussed in the following section, which examines the potential role of RAAS activation in endothelial dysfunction, oxidative stress, inflammation, microvascular injury, and target organ damage during hypertensive emergencies [69,70,71,72].

### 3.4. Pathophysiological Role of the Renin–Angiotensin–Aldosterone System in Hypertensive Emergencies

The pathophysiology of hypertensive emergencies is multifactorial and extends beyond the abrupt elevation of arterial blood pressure. Although excessive mechanical stress on the vascular wall may initiate endothelial injury, the subsequent progression to widespread target organ damage appears to be amplified by maladaptive neurohormonal activation [54]. Among the various regulatory systems involved, the RAAS has been proposed as an important contributor to vascular dysfunction, potentially integrating hemodynamic, inflammatory, oxidative, immune, and thrombotic responses [56]. Rather than functioning solely as a regulator of systemic blood pressure, the RAAS may control molecular events that contribute to endothelial dysfunction, loss of vascular autoregulation, microvascular injury, and progressive organ damage during hypertensive emergencies [55,56,57,58,59,73].

Experimental and clinical evidence suggests that RAAS activation can occur rapidly following acute elevations in blood pressure and may contribute to the amplification of vascular injury through multiple interconnected mechanisms [45,46,47,48]. Activation of the classical ACE/angiotensin II (Ang II)/AT1 receptor axis stimulates vasoconstriction, enhances sympathetic nervous system activity, increases oxidative stress, activates inflammatory signaling pathways, promotes coagulation, and induces structural alterations within the vascular wall [15,16,17]. Importantly, these biological responses are thought to represent independent mechanisms capable of perpetuating tissue injury even after systemic hemodynamic stabilization, although their precise contribution to hypertensive emergencies remains to be fully defined [49,50,51,52].

### 3.5. Angiotensin II and Acute Vasoconstriction

Angiotensin II is the central effector peptide of the classical RAAS and exerts profound effects on vascular homeostasis through activation of the AT1 receptor [1]. During hypertensive emergencies, excessive Ang II production is believed to result in intense systemic vasoconstriction, mediated by increased intracellular calcium influx into vascular smooth muscle cells [1,2]. This response may rapidly elevate systemic vascular resistance, increase left ventricular afterload, reduce tissue perfusion, and aggravate arterial hypertension [43,44,45]. Beyond its direct contractile effects, Ang II has been shown to potentiate sympathetic neurotransmission by stimulating norepinephrine release and inhibiting its reuptake [15,16,17]. Increased sympathetic activation may further enhance vasoconstriction, tachycardia, myocardial oxygen demand, and renin secretion, establishing a positive feedback loop that sustains RAAS activation [2,3]. In addition, Ang II appears to stimulate vasopressin release and activate central autonomic nuclei involved in cardiovascular regulation, reinforcing neurohormonal dysregulation during acute hypertensive crises [36,37,38,39]. Persistent vasoconstriction may compromise regional tissue perfusion despite elevated systemic arterial pressure, contributing to ischemia in organs such as the brain, kidneys, and myocardium [50,51].

### 3.6. Endothelial Dysfunction

Endothelial dysfunction is widely recognized as one of the earliest and most critical events in hypertensive emergencies [70]. Under physiological conditions, endothelial cells regulate vascular tone, coagulation, inflammation, permeability, and leukocyte trafficking. Acute RAAS activation is thought to disrupt these functions [42,43,44]. Angiotensin II has been shown to reduce eNOS activity and increase nitric oxide degradation through excessive ROS production [1,2,70]. Reduced nitric oxide bioavailability may impair vasodilation, promote vascular stiffness, facilitate platelet aggregation, and enhance leukocyte adhesion. At the same time, Ang II stimulates endothelin-1 synthesis, producing potent vasoconstrictor responses [15,16,17,35,36,37,38].

Endothelial activation may also induce the expression of adhesion molecules, such as VCAM-1, ICAM-1, E-selectin, and P-selectin, promoting leukocyte recruitment. This inflammatory phenotype contributes to vascular permeability, tissue edema, and microvascular injury. Disruption of the endothelial glycocalyx, described in acute cardiovascular disorders, has been suggested as an additional mechanism that may accelerate vascular injury during hypertensive emergencies [70].

### 3.7. Oxidative Stress

Oxidative stress is considered one of the principal mechanisms through which RAAS activation may mediate vascular injury. AT1 receptor stimulation activates enzymatic sources of ROS, particularly NADPH oxidases (NOX1–NOX5), which generate superoxide within the vascular wall [15,16,17,70,71,72,73,74]. Excessive ROS production reacts with nitric oxide to form peroxynitrite, reducing nitric oxide bioavailability and producing reactive molecules that can damage proteins, lipids, DNA, and mitochondria. Mitochondrial dysfunction may further amplify ROS generation, creating a cycle of oxidative injury. Oxidative stress also activates redox-sensitive transcription factors, such as NF-κB, AP-1, and HIF-1α, which regulate inflammatory mediators [2,70]. Although much of this evidence comes from experimental and chronic hypertension studies, oxidative stress markers have been reported in hypertensive emergencies, suggesting a contributory role [70,71,72,73,74].

### 3.8. Inflammation and Immune Activation

Hypertensive emergencies are increasingly viewed as inflammatory vascular disorders rather than purely hemodynamic events [75,76,77,78,79,80]. Angiotensin II has been shown in experimental studies to activate NF-κB signaling, increase transcription of pro-inflammatory cytokines (IL-6, TNF-α, IL-1β, MCP-1), and stimulate the NLRP3 inflammasome [81,82,83,84,85]. These mechanisms promote leukocyte recruitment and immune cell infiltration into the vascular wall, perpetuating the injury. While most evidence is derived from chronic hypertension and animal models, limited clinical observations suggest that inflammatory activation may also accompany acute vascular damage in hypertensive emergencies [41,42,43,44,86]. The involvement of Th17 cells and regulatory T-cell imbalance, as described in hypertension-associated vascular inflammation, represents a plausible mechanism in acute crises but requires further validation.

### 3.9. Microvascular Injury and Thrombotic Activation

Failure of microvascular integrity is a pathological feature of hypertensive emergencies. Excessive mechanical stress combined with RAAS-induced endothelial dysfunction may result in disruption of the endothelial barrier, increased vascular permeability, fibrinoid necrosis, and platelet activation [45,46,47,70]. Exposure of subendothelial collagen initiates platelet adhesion and activation of the coagulation cascade, whereas endothelial production of tissue factor further enhances thrombin generation. Microvascular thrombosis subsequently compromises tissue perfusion and contributes to ischemic injury, despite persistently elevated systemic blood pressure. Histopathological examination frequently demonstrates fibrinoid necrosis of small arteries and arterioles, endothelial swelling, fragmented erythrocytes, and thrombotic microangiopathy, particularly in the kidneys and retina [48,49,50,51].

In addition to these well-established pathological features, emerging evidence suggests that neutrophil extracellular traps (NETs), complement activation, and endothelial microparticles may participate in thromboinflammatory responses associated with severe hypertension. However, their contributions to hypertensive emergencies remain unclear and require further investigation [52,53,54].

### 3.10. RAAS-Mediated Target Organ Damage

The cumulative effects of vasoconstriction, oxidative stress, endothelial dysfunction, inflammation, and microvascular thrombosis ultimately result in acute target organ injury. In the brain, excessive Ang II signaling contributes to blood–brain barrier disruption, cerebral edema, impaired autoregulation, and neuroinflammation, facilitating hypertensive encephalopathy, PRES, ischemic stroke, and intracerebral hemorrhage [55,56,57,58]. Within the heart, RAAS activation increases myocardial oxygen demand through elevated afterload while simultaneously impairing coronary microvascular perfusion. These mechanisms contribute to acute heart failure, myocardial ischemia, ventricular dysfunction, and malignant arrhythmias observed during hypertensive emergencies [59,60,61,62]. In the kidneys, excessive intrarenal RAAS activation promotes afferent and efferent arteriolar vasoconstriction, glomerular hypertension, tubular ischemia, podocyte injury, and inflammation. These alterations accelerate acute kidney injury and may lead to persistent deterioration of renal function despite successful blood pressure control [2,63,64,65,66].

Retinal vascular injury mirrors systemic microvascular dysfunction and is characterized by endothelial damage, retinal hemorrhage, cotton-wool spots, hard exudates, and papilledema. As retinal vessels are readily accessible for direct visualization, hypertensive retinopathy provides valuable insights into the severity of systemic endothelial injury during hypertensive emergencies [67,68,69,87,88,89,90]. Collectively, these findings demonstrate that the RAAS may function as an important biological network linking acute blood pressure elevation to endothelial dysfunction, oxidative stress, inflammation, microvascular injury, and target organ damage. This integrated pathophysiological framework provides a rationale for investigating circulating and tissue RAAS biomarkers and exploring novel therapeutic strategies aimed at modulating specific components of the system in patients with hypertensive emergencies [15,16,17,70].

**Figure 1:**
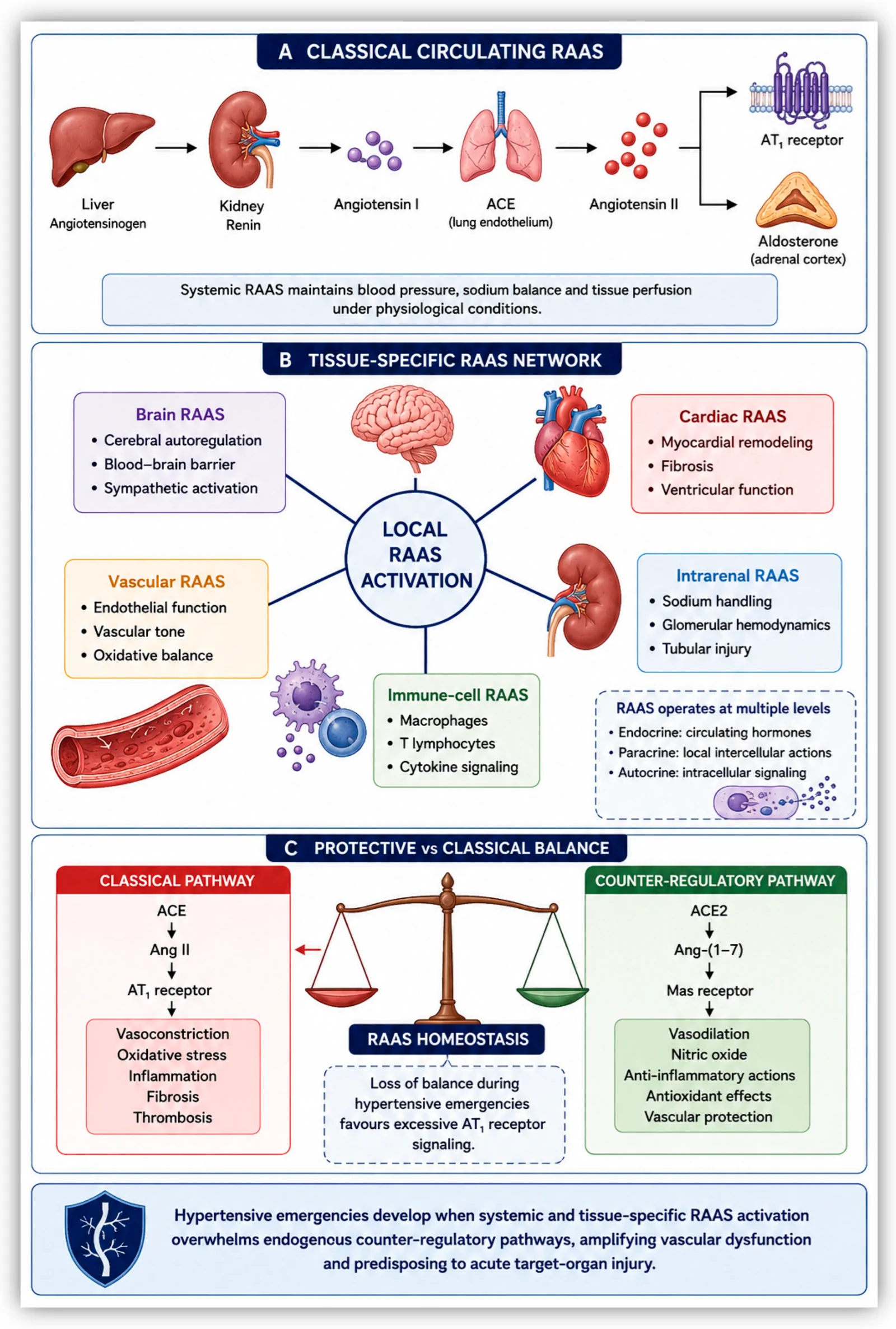
Systemic and tissue-specific organization of the renin–angiotensin–aldosterone system (RAAS) and its relevance to hypertensive emergencies. The RAAS functions as an integrated endocrine, paracrine, autocrine, and tissue-specific signaling network. The classical circulating pathway begins with renin release from the kidney, leading to the generation of angiotensin II and activation of AT1 receptors, whereas aldosterone contributes to sodium retention and cardiovascular regulation. In parallel, local RAAS systems operate within the brain, heart, kidneys, vasculature, and immune cells, regulating organ-specific physiological and pathological responses independently of circulating hormone levels. The balance between the classical ACE/Ang II/AT1 receptor axis and the protective ACE2/Ang-(1–7)/Mas receptor axis is essential for maintaining vascular homeostasis. During hypertensive emergencies, excessive activation of the classical pathway may overwhelm endogenous counter-regulatory mechanisms, predisposing patients to endothelial dysfunction, vascular injury, and acute target-organ damage. Abbreviations: RAAS, renin–angiotensin–aldosterone system; ACE, angiotensin-converting enzyme; ACE2, angiotensin-converting enzyme 2; Ang II, angiotensin II; Ang-(1–7), angiotensin-(1–7); AT1 receptor, angiotensin II type 1 receptor; Mas receptor, Mas-related G-protein-coupled receptor.

The organization of the RAAS in hypertensive emergencies is illustrated in Figure 1, highlighting the interplay between systemic and tissue-specific RAAS activation. The schematic emphasizes how the classical circulating pathway (ACE/Ang II/AT1 receptor axis) promotes vasoconstriction, oxidative stress, inflammation, and fibrosis, whereas the counter-regulatory ACE2/Ang-(1–7)/Mas receptor axis provides vasodilatory and protective effects. Importantly, the figure also depicts local RAAS networks within the brain, heart, kidneys, vasculature, and immune cells, underscoring that organ-specific activation contributes to vascular dysfunction and acute target-organ injury beyond blood pressure elevation.

### 3.11. Clinical Evidence Linking RAAS to Hypertensive Emergencies

Clinical evidence linking RAAS dysregulation to hypertensive emergencies has expanded considerably in recent years but remains substantially less robust than the experimental evidence. Most available data are derived from single-center observational studies, retrospective cohorts, pharmacogenetic analyses, and emerging multicenter registries rather than randomized clinical trials. Nevertheless, these investigations consistently support an association between RAAS activation, endothelial dysfunction, and hypertension-mediated organ damage (HMOD), suggesting that early pharmacological interruption of RAAS may improve renal outcomes in selected patients (Table 1) [1,2,62,67,69,70,71,90].

Among the most informative studies, Endo et al. retrospectively evaluated patients hospitalized with hypertensive emergencies complicated by multiple organ dysfunctions in a retrospective cohort. Patients who received early initiation of RAS inhibitors experienced significantly greater recovery of estimated glomerular filtration rate (eGFR) and superior two-year renal survival than those treated primarily with calcium channel blockers. These findings support the hypothesis that RAAS activation contributes to sustained renal injury rather than merely being a secondary response to acute blood pressure elevation. However, the retrospective design, relatively small sample size, and absence of serial neurohormonal measurements preclude causal inferences [71].

Complementing these observations, Miyake et al. directly evaluated the circulating RAAS components at the time of hospital admission. Elevated plasma aldosterone concentrations were independently associated with a greater burden of hypertension-mediated organ damage, particularly among patients presenting with involvement of three or more organs, whereas plasma renin activity showed weaker associations. These findings suggest that mineralocorticoid activation may be a more relevant determinant of acute organ injury than upstream RAAS activation. Nevertheless, biomarker assessment was limited to a single time point, preventing the evaluation of the temporal dynamics of neurohormonal activation during the acute phase [69].

Additional support for the pathogenic role of RAAS was provided by Ueno et al., who reported that early pharmacological renin inhibition was associated with improved long-term preservation of renal function following hypertensive emergencies [1]. Together with the findings of Endo et al., these observations suggest that early interruption of RAAS signaling may attenuate persistent renal injury after an acute event. However, both investigations were retrospective, conducted at single centers, and lacked standardized serial assessments of circulating RAAS biomarkers, limiting mechanistic interpretation [1,2,66,71].

Evidence supporting endothelial dysfunction as a downstream consequence of neurohormonal activation was provided by Sansone et al., who demonstrated significantly increased circulating endothelial microparticles in patients with hypertensive emergencies compared with those with uncomplicated hypertension and healthy controls. The observed increase in endothelial activation markers reinforces the concept that severe hypertension is accompanied by acute endothelial injury and vascular activation. However, this mechanistic study did not directly quantify RAAS activity and therefore could not establish a causal relationship between neurohormonal activation and endothelial dysfunction [70].

Additional evidence linking the RAAS to hypertensive emergencies derives from a pharmacogenetic study by Vilela-Martin et al., which demonstrated that the antihypertensive response to ACE inhibition varied according to the angiotensin-converting enzyme insertion/deletion polymorphism. Patients with the DD genotype exhibited greater responsiveness to ACE inhibition, suggesting that genetic variability within the RAAS may contribute to interindividual differences in disease severity and therapeutic response. Although these findings support the biological relevance of the RAAS in hypertensive emergencies, the study evaluated genetic susceptibility rather than circulating neurohormonal activation during acute events [90].

Recently, multicenter clinical registries have substantially expanded the clinical characterization of hypertensive emergencies. The ERIDANO registry demonstrated the marked heterogeneity of hypertensive emergency phenotypes across participating centers and provided an important infrastructure for future biomarker-based investigations [62]. Likewise, prospective studies by Fragoulis et al. confirmed that the extent of hypertension-mediated organ damage is the principal determinant of subsequent cardiovascular prognosis. Although neither study directly evaluated RAAS activation, they established an essential clinical framework for future investigations integrating neurohormonal biomarkers with standardized phenotyping and long-term outcomes [67].

Collectively, current clinical evidence supports an association between RAAS activation, aldosterone excess, endothelial dysfunction, genetic variability, and hypertension-induced organ damage. However, the available literature remains largely observational and is limited by heterogeneous patient populations, modest sample sizes, retrospective or cross-sectional designs, and non-standardized biomarker assessment protocols. Importantly, most studies measured circulating RAAS components only after hospital admission, making it difficult to determine whether neurohormonal activation precedes organ injury or represents a secondary response to acute hemodynamic stress. Consequently, no RAAS-related biomarker currently demonstrates sufficient diagnostic or prognostic performance to guide the routine clinical management of hypertensive emergencies. Future large multicenter prospective studies incorporating serial measurements of renin, angiotensin II, aldosterone, and endothelial injury biomarkers are essential to define the temporal dynamics and clinical significance of RAAS activation and to identify patients most likely to benefit from targeted neurohormonal interventions [1,2,62,67,69,70,71,90].

### 3.12. Renin and Aldosterone Activity in Hypertensive Emergencies

Plasma renin activity and aldosterone concentration have traditionally been used as indirect markers of RAAS activation [1]. In chronic hypertension, RAAS phenotypes vary considerably, with some individuals exhibiting low-renin hypertension and others demonstrating increased renin activity [2]. During hypertensive emergencies, acute sympathetic activation, renal ischemia, and impaired autoregulation may stimulate renin secretion, contributing to increased Ang II formation and aldosterone release [73,74]. Elevated aldosterone levels may be particularly relevant in patients with severe hypertension complicated by cardiovascular and renal injuries. In addition to sodium retention, aldosterone promotes vascular inflammation, endothelial dysfunction, oxidative stress, myocardial fibrosis, and renal injury through mineralocorticoid receptor activation [1,2,3].

Although limited in size and heterogeneous in design, clinical studies have reported an association between aldosterone excess and increased cardiovascular risk [75,76,77]. However, the interpretation of circulating renin and aldosterone levels during acute hypertensive emergencies is challenging. Many patients receive diuretics, RAAS blockers, beta-blockers, or vasodilators before laboratory evaluation, which may substantially modify the measured hormone concentrations. Thus, circulating biomarkers may not fully reflect the tissue-specific activation responsible for acute organ injury [78].

### 3.13. Angiotensin II and Tissue RAAS Activation

The direct measurement of Ang II has historically been limited by its rapid degradation, short plasma half-life, and methodological variability [1,2,3]. Nevertheless, translational studies suggest that excessive Ang II signaling contributes to endothelial dysfunction and vascular injury in severe hypertensive states [79]. Importantly, tissue-specific RAAS activation has changed the interpretation of the clinical RAAS measurements [2]. Circulating Ang II concentrations may underestimate local activation within the vascular wall, kidneys, heart, and brain [2,4,66].

Intrarenal Ang II accumulation is associated with impaired autoregulation, sodium retention, inflammation, and progression of kidney injury [2]. These mechanisms are highly relevant in hypertensive emergencies, in which acute renal dysfunction is among the most frequent manifestations of target organ damage [80,81,82]. Similarly, vascular RAAS activation may contribute to endothelial dysfunction, increased arterial stiffness, and impaired microcirculatory regulation [66]. Studies using imaging, molecular biomarkers, and vascular function assessments suggest that patients with severe hypertension exhibit endothelial activation and oxidative stress. However, such findings should not be interpreted automatically as proof of excessive Ang II signaling, as multiple pathways can produce similar biomarker profiles [70,83,84].

### 3.14. ACE2 and the Protective RAAS Axis

The ACE2/Ang-(1–7)/Mas receptor pathway has emerged as an important counter-regulatory mechanism in cardiovascular diseases. Reduced ACE2 activity or impaired conversion of Ang II into Ang-(1–7) may contribute to an imbalance favoring AT1 receptor-mediated injury [1,2,3]. However, clinical evidence specifically addressing ACE2 activity in hypertensive emergencies remains scarce. Most available data are extrapolated from studies on cardiovascular and renal diseases, where reduced ACE2 expression has been associated with endothelial dysfunction, inflammation, fibrosis, and adverse outcomes [8,15,16,17,70].

### 3.15. RAAS Biomarkers and Target-Organ Damage

An emerging area of investigation involves the identification of RAAS-related biomarkers that can predict acute organ injury and clinical outcomes. Urinary angiotensinogen has received particular attention as a marker of intrarenal RAAS activation and is associated with renal inflammation, proteinuria, and progression of kidney disease [2,66,77]. Other potential biomarkers include inflammatory mediators, oxidative stress markers, endothelial injury molecules, and extracellular vesicle-associated RAAS components. Integrating these biomarkers with clinical parameters may improve risk stratification and allow the identification of patients at a higher risk of persistent organ dysfunction after hypertensive emergencies [78].

However, no RAAS biomarker has demonstrated sufficient clinical utility to guide acute management decisions. Most available studies are small, heterogeneous in design, and often confounded by prior antihypertensive therapy, phenotype variability, and timing of sample collection. Therefore, evidence of endothelial activation or oxidative stress should be interpreted cautiously, as these findings are not specific for excessive Ang II signaling and may reflect overlapping pathways of vascular injury.

Table 2 summarizes the principal RAAS-related biomarkers investigated in human and translational studies, the clinical populations in which they were evaluated, reported associations with acute organ injury, analytical limitations, and current clinical applicability. The evidence remains preliminary, with most biomarkers lacking validation for guide acute management decisions.

### 3.16. Therapeutic Implications of RAAS Modulation in Hypertensive Emergencies

The management of hypertensive emergencies requires immediate but controlled reduction in blood pressure to limit ongoing organ injury while avoiding excessive lowering that may compromise tissue perfusion [1,2]. Current international guidelines, including those from the European Society of Hypertension and the American Heart Association, recommend the use of rapidly acting intravenous antihypertensive agents, such as calcium channel blockers, vasodilators, and beta-blockers, selected according to the affected organ system and the specific clinical presentation [73,74].

Although RAAS activation may play a central role in the pathophysiology of hypertensive emergencies, direct RAAS blockade is not currently considered the first-line therapy during the acute phase. This approach contrasts with chronic hypertension management, where ACE inhibitors, angiotensin receptor blockers (ARBs), and mineralocorticoid receptor antagonists are fundamental components of cardiovascular and renal protection [1,2,3].

A Brazilian case–control study evaluated the genetic and pharmacological factors associated with hypertensive emergencies. The investigators reported that patients experiencing hypertensive crises had a lower frequency of the ACE II genotype than controls, and that limited use of RAAS-blocking agents (such as ACE inhibitors or ARBs) was associated with an increased risk of emergency presentation. The principal finding was that genetic predisposition combined with reduced exposure to RAAS blockade may contribute to susceptibility to hypertensive emergencies in patients with CKD. However, this study was relatively small, focused on a single population, and subject to confounding by prior treatment patterns, which limits the generalizability of its conclusions [90]. These observations highlight the potential role of RAAS modulation and genetic variability in determining risk; however, they do not alter current international recommendations, which emphasize that acute therapy should be guided primarily by the affected organ system and clinical presentation.

### 3.17. ACE Inhibitors and Angiotensin Receptor Blockers

ACE inhibitors and ARBs are well-established therapies for chronic hypertension, with robust evidence demonstrating improvements in endothelial function, vascular remodeling, and cardiovascular outcomes [1,2,66]. However, their role in hypertensive emergencies remains unclear. Limitations include delayed onset of action, potential hemodynamic instability, and the risk of excessive reduction in glomerular filtration, particularly in patients with acute kidney injury or bilateral renal artery stenosis [71]. Current international recommendations do not support their use as first-line agents in acute settings [73,74]. Nevertheless, the early reintroduction of RAAS blockers after stabilization may provide long-term benefits by reducing inflammation, fibrosis, and cardiovascular remodeling. Determining the optimal timing for restarting these therapies remains an important clinical question, supported mainly by observational data [1,2,3].

### 3.18. Mineralocorticoid Receptor Antagonists

Mineralocorticoid receptor antagonists (MRAs), such as spironolactone and eplerenone, are established treatments for resistant hypertension and heart failure, with strong evidence of benefits in chronic diseases [1,2,15,16,17]. Their effects extend beyond blood pressure reduction and include the attenuation of oxidative stress, inflammation, endothelial dysfunction, and fibrosis. Patients with hyperaldosteronism or resistant hypertension may represent a subgroup in which mineralocorticoid receptor activation contributes substantially to hypertensive emergencies [15,16,17]. Evidence in acute crises is limited, and prospective studies are needed to evaluate whether the early identification of aldosterone-driven phenotypes can improve individualized treatment strategies [71].

Novel therapeutic strategies targeting specific RAAS components are currently under investigation. These include the enhancement of ACE2 activity, administration of Ang-(1–7) analogues, Mas receptor agonists, selective AT1 receptor modulators, and therapies targeting intracellular RAAS signaling pathways. Although promising in experimental and translational models, these approaches have not yet demonstrated safety or efficacy in patients with hypertensive emergencies. Therefore, they should be considered experimental and not clinically applicable at present. The major challenge remains the selective inhibition of harmful RAAS signaling while preserving the physiological functions necessary for cardiovascular adaptation [90].

Table 3 summarizes the major RAAS mechanisms involved in the hypertensive emergency.

## 4. Emerging Concepts in RAAS Biology and Hypertensive Emergencies

Recent advances in cardiovascular biology have expanded the understanding of the RAAS from a classical endocrine system to a complex regulatory network involving multiple tissues, intracellular pathways, immune responses, and molecular interactions [70,71,72,73]. These emerging concepts may help explain the heterogeneity observed among patients with hypertensive emergencies and could inform future precision-based therapeutic strategies [74,75,76,77]. However, most of these mechanisms remain speculative and require further validation in the context of acute hypertensive crises.

### 4.1. Intracellular RAAS and Non-Classical Angiotensin Signaling

The discovery of intracellular RAAS components has challenged the traditional concept that angiotensin peptides act only through membrane-bound receptors. Experimental studies have demonstrated intracellular Ang II generation and signaling in vascular smooth muscle cells, cardiomyocytes, renal tubular cells, and immune cells [1,2,3,4]. These pathways may contribute to persistent cellular responses even after a decline in extracellular Ang II levels, potentially explaining why tissue injury can continue despite blood pressure reduction [63,64,65]. The clinical relevance of intracellular RAAS activation in hypertensive emergencies remains largely unexplored, and current evidence should be considered hypothesis-generating rather than established [2,3,4,5].

### 4.2. Immune System Activation and RAAS Crosstalk

Hypertension and target organ damage are closely associated with immune dysregulation [47]. RAAS interacts with innate and adaptive immune pathways, creating a bidirectional relationship in which Ang II promotes inflammation, while inflammatory mediators further stimulate RAAS activation [48,73]. Imbalances between Th17 and regulatory T cells have been implicated in vascular injury [49,66,67,68]. In hypertensive emergencies, immune–RAAS interactions may amplify vascular damage; however, direct clinical evidence is limited. Although these concepts remain speculative, they highlight potential avenues for future immunomodulatory strategies [70].

### 4.3. RAAS, Extracellular Vesicles, and Intercellular Communication

Extracellular vesicles (EVs) have emerged as mediators of cardiovascular communication, transporting proteins, lipids, and microRNAs. Some studies have suggested that EVs may contain RAAS-related molecules [1,2]. In hypertensive emergencies, endothelial-derived EVs could contribute to vascular inflammation and coagulation; however, current evidence is preliminary and largely extrapolated from other cardiovascular disorders [69,70]. Their role should be regarded as an emerging research direction rather than an established one.

### 4.4. Gut Microbiota and RAAS Regulation

Gut microbiota have been linked to hypertension through inflammation, immune activation, and metabolite production [1,2,3,4]. Dysbiosis may influence RAAS activity and vascular function; however, direct evidence in hypertensive emergencies is lacking. This area remains speculative, and future studies are needed to determine whether microbiome-targeted interventions could reduce susceptibility to acute hypertensive complications [71,72].

### 4.5. Artificial Intelligence and Precision Medicine Approaches

Artificial intelligence (AI) and machine learning may integrate clinical variables, imaging, biomarkers, and genomic data to identify patients at risk of severe target-organ injury [1]. Precision medicine approaches could classify patients into biological phenotypes (e.g., Ang II-driven, aldosterone-mediated, inflammatory, or sympathetic activation). Although promising, these strategies remain conceptual and require validation in large prospective cohorts before clinical application in hypertensive emergencies [37,88,89].

## 5. Current Knowledge Gaps and Future Directions

Despite substantial advances in understanding RAAS biology, several important questions regarding its role in hypertensive emergencies remain unanswered. One major gap concerns the temporal dynamics of RAAS activation during acute crisis. It is unclear whether RAAS activation is an initiating event, a consequence of vascular injury, or a self-perpetuating mechanism that drives disease progression. Current evidence is largely experimental or observational, with most studies evaluating biomarkers at a single time point only. Large prospective multicenter studies with serial biomarker measurements are needed to clarify these temporal patterns.

Another unresolved issue is the clinical relevance of circulating RAAS biomarkers in patients with obesity. Plasma renin activity, aldosterone, Ang II, and ACE2 levels may not accurately reflect tissue-specific RAAS activation, thereby limiting their diagnostic and prognostic utility. Available studies are small, heterogeneous, and often confounded by prior antihypertensive therapy, variability in disease phenotype, and the timing of sample collection. Standardized biomarker protocols and their correlation with clinically meaningful outcomes are essential for determining their value in acute management.

The therapeutic role of RAAS modulation in hypertensive emergencies remains unclear. Although chronic RAAS inhibition provides well-established cardiovascular and renal protection, evidence supporting acute RAAS-directed therapy is insufficient. Randomized clinical trials are lacking, and most data are from retrospective or observational studies. Future trials should evaluate whether RAAS-targeted interventions can reduce mortality, accelerate organ recovery, and prevent long-term complications.

Additional knowledge gaps exist in non-classical RAAS pathways. The roles of ACE2, Ang-(1–7), Mas receptor signaling, intracellular RAAS components, and immune-related RAAS activation in acute hypertensive injury remain incompletely understood. Current evidence is mostly experimental or extrapolated from studies on chronic cardiovascular and renal diseases. Translational studies integrating tissue samples, molecular profiling, and clinical phenotyping are needed to clarify the relevance of these findings in hypertensive emergencies.

Finally, the current classification systems for hypertensive emergencies are based primarily on clinical manifestations and affected organs, rather than molecular mechanisms. Incorporating biological phenotyping may improve patient stratification and allow for personalized treatment strategies. Large prospective studies combining genomic, transcriptomic, proteomic, metabolomic, and clinical data could identify distinct RAAS-driven phenotypes, such as Ang II-mediated hypertension, aldosterone excess, impaired ACE2 activity, inflammatory activation, and oxidative stress predominance. Advanced molecular technologies, including single-cell RNA sequencing and spatial transcriptomics, may provide unprecedented insights into tissue-specific RAAS activation and reveal novel therapeutic targets.

## 6. Conclusions

Hypertensive emergencies are complex vascular syndromes in which acute blood pressure elevation interacts with profound molecular and cellular disturbances. Although increased mechanical stress contributes to endothelial injury, activation of the renin–angiotensin–aldosterone system is considered a potentially important mediator linking hypertension to oxidative stress, inflammation, vascular dysfunction, microvascular injury, and target organ damage. The classical ACE/angiotensin II/AT1 receptor pathway is associated with vasoconstriction, endothelial dysfunction, inflammatory activation, fibrosis, and tissue injury, whereas the ACE2/angiotensin-(1–7)/Mas receptor axis appears to provide counter-regulatory protective effects. Disruption of this balance may significantly contribute to the pathogenesis of hypertensive emergencies. Despite major advances in RAAS biology, important clinical questions remain regarding biomarker utility, the timing of RAAS activation, and therapeutic modulation during acute hypertensive states. Although blood pressure reduction remains the cornerstone of managing hypertensive emergencies, emerging research highlights the potential relevance of molecular profiling, precision medicine, and RAAS-targeted strategies. At present, these approaches have limited direct clinical application; however, future studies may clarify whether they could complement conventional therapy by enabling more individualized management. A deeper understanding of RAAS-mediated mechanisms may ultimately support improved risk prediction, earlier recognition of target organ injury, and the development of innovative therapies capable of reducing the substantial burden associated with hypertensive emergencies.

## Figures and Tables

**Table 1:** Principal human studies evaluating RAAS-related mechanisms in hypertensive emergencies.

Study	Sample Size	Clinical Phenotype	RAAS Assessment	Main Findings	Principal Limitations
Endo et al., 2023 [2]	49 patients	Hypertensive emergency with multiple organ dysfunction	Early initiation of ACE inhibitor/ARB	Early RAAS blockade associated with greater eGFR recovery and improved two-year renal survival	Retrospective; non-randomized; single center; no serial biomarker assessment
Fragoulis et al., 2023 [67]	97 patients	Hypertensive emergency	Clinical outcomes according to HMOD burden	Greater extent of organ damage associated with worse 12-month cardiovascular prognosis	No direct RAAS biomarker assessment
Miyake et al., 2024 [69]	63 patients	Hypertensive emergency	Plasma renin activity and aldosterone	Aldosterone independently associated with greater burden of hypertension-mediated organ damage; weaker association for plasma renin activity	Cross-sectional; single-center; single biomarker measurement
Sansone et al., 2018 [70]	41 participants	Hypertensive emergency	Endothelial microparticles (CD31+/41−, CD62E+, CD144+)	Marked endothelial activation and increased endothelial microparticle release during hypertensive emergencies	Pilot study; mechanistic design; no direct RAAS biomarkers
Ueno et al., 2024 [1]	20 patients	Hypertensive emergency	Early renin inhibition	Early renin inhibition associated with improved long-term preservation of renal function	Retrospective; observational; single-center
Vallelonga et al., 2023 [62]	122 patients	Hypertensive emergencies and urgencies	Clinical registry	Demonstrated marked clinical heterogeneity and established a multicenter platform for future biomarker studies	Preliminary registry; no direct RAAS measurements
Vilela-Martin et al., 2013 [90]	118 patients	Hypertensive emergency	ACE insertion/deletion polymorphism	DD genotype associated with greater blood pressure reduction after RAAS-blocking agents, suggesting genetic influence on RAAS responsiveness	Small pharmacogenetic study; no circulating RAAS biomarkers; single center

Abbreviations: ACE, angiotensin-converting enzyme; ARB, angiotensin II receptor blocker; DD, deletion/deletion genotype; eGFR, estimated glomerular filtration rate; HMOD, hypertension-mediated organ damage; RAAS, renin–angiotensin–aldosterone system; CD, Cluster of Differentiation; CD31, Cell Adhesion Molecule-1; CD41, Integrin αIIb (GPIIb); CD62E, E-selectin; CD144, Vascular Endothelial Cadherin (VE-cadherin).

**Table 2:** Proposed RAAS-related biomarkers and their associations with target-organ damage in hypertensive emergencies.

Biomarker	Clinical Population Studied	Association with Organ Injury	Analytical Limitations	Clinical Applicability
Urinary angiotensinogen [1,2,66]	Patients with severe hypertension, CKD	Linked to proteinuria, renal inflammation, progression of kidney disease	Influenced by renal function, assay variability	Potential marker of intrarenal RAAS activation; not validated for acute use
Plasma renin activity [1,2,15,16,17]	Hypertensive emergencies, malignant hypertension	Associated with acute BP elevation and renal ischemia	Altered by prior RAAS blockers, diuretics, timing of measurement	Limited utility in acute setting
Aldosterone [1,2,15,16,17]	Severe hypertension with CV/renal injury	Correlated with target-organ damage, fibrosis, inflammation	Highly variable; affected by medications and posture	May indicate mineralocorticoid-driven injury; not yet actionable
Oxidative stress markers (ROS, NO metabolites) [70]	Severe hypertension, acute crises	Associated with endothelial dysfunction and vascular injury	Non-specific; multiple pathways produce similar changes	Supportive evidence of vascular stress; not specific for RAAS
Endothelial injury molecules (VCAM-1, ICAM-1, glycocalyx fragments)	Acute hypertensive syndromes	Linked to vascular permeability, edema, microvascular injury	Assay standardization lacking	Promising but experimental
Extracellular vesicle RAAS components [70]	Translational studies, pilot cohorts	Suggest involvement in thromboinflammation	Technical complexity, limited reproducibility	Research tool; not clinical

Abbreviations: RAAS, renin–angiotensin–aldosterone system; CKD, chronic kidney disease; BP, blood pressure; CV, cardiovascular; ROS, reactive oxygen species; NO, nitric oxide; VCAM-1, vascular cell adhesion molecule-1; ICAM-1, intercellular adhesion molecule-1.

**Table 3:** Major RAAS mechanisms involved in hypertensive emergencies.

RAAS Component	Molecular Mechanism	Consequence in Hypertensive Emergencies	Supporting Evidence Type
Renin [1,2,15,16,17]	Increased activation due to renal ischemia and sympathetic stimulation	Enhanced Ang II production	Experimental, translational
Angiotensin II [1,2,15,16,17]	Central effector peptide; stimulates multiple pathways	Vasoconstriction, sympathetic activation, promotion of oxidative stress and inflammation	Experimental, clinical
AT1 receptor [1,2,15,16,17]	Receptor-mediated signaling of Ang II	Vascular injury, remodeling, pro-inflammatory and pro-thrombotic responses	Experimental, translational
Aldosterone [1,2,15,16,17]	Mineralocorticoid hormone; stimulates sodium retention	Endothelial dysfunction, fibrosis, renal injury	Translational, clinical
Mineralocorticoid receptor [1,2,15,16,17]	Activation by aldosterone	Oxidative stress, inflammation, fibrosis, sodium retention	Experimental, translational
ACE [1,2,15,16,17]	Conversion of Ang I into Ang II	Amplification of classical RAAS pathway	Experimental
ACE2 [1,2,15,16,17]	Degradation of Ang II into Ang-(1–7)	Protective vascular effects	Experimental, translational
Ang-(1–7) [1,2,15,16,17]	Mas receptor activation	Vasodilation, antioxidant and anti-inflammatory effects	Experimental
Mas receptor [1,2,15,16,17]	Counter-regulatory signaling	Tissue protection	Experimental

Abbreviations: RAAS, renin–angiotensin–aldosterone system; ACE, angiotensin-converting enzyme; ACE2, angiotensin-converting enzyme 2; Ang I, angiotensin I; Ang II, angiotensin II; Ang-(1–7), angiotensin-(1–7); AT1 receptor, angiotensin II type 1 receptor; Mas receptor, Mas proto-oncogene receptor.

## Data Availability

No new data were created or analyzed in this study. Data sharing is not applicable to this article.

## Abbreviations

The following abbreviations are used in this manuscript:

ACE	Angiotensin-Converting Enzyme
ACE2	Angiotensin-Converting Enzyme 2
AI	Artificial Intelligence
Ang I	Angiotensin I
Ang II	Angiotensin II
Ang-(1–7)	Angiotensin-(1–7)
AP-1	Activator Protein-1
ARB	Angiotensin Receptor Blocker
AT1R	Angiotensin Type 1 Receptor
DNA	Deoxyribonucleic Acid
eNOS	Endothelial Nitric Oxide Synthase
HIF-1α	Hypoxia-Inducible Factor-1 Alpha
ICAM-1	Intercellular Adhesion Molecule-1
IL-1β	Interleukin-1 Beta
IL-6	Interleukin-6
IL-18	Interleukin-18
JAK/STAT	Janus Kinase/Signal Transducer and Activator of Transcription
MAPKs	Mitogen-Activated Protein Kinases
MCP-1	Monocyte Chemoattractant Protein-1
MeSH	Medical Subject Headings
NADPH	Nicotinamide Adenine Dinucleotide Phosphate
NETs	Neutrophil Extracellular Traps
NF-κB	Nuclear Factor Kappa B
NLRP3	NLR Family Pyrin Domain Containing 3
NOX	NADPH Oxidase
NOX1	NADPH Oxidase 1
NOX2	NADPH Oxidase 2
NOX4	NADPH Oxidase 4
NOX5	NADPH Oxidase 5
PRES	Posterior Reversible Encephalopathy Syndrome
RAAS	Renin–Angiotensin–Aldosterone System
RNA	Ribonucleic Acid
ROS	Reactive Oxygen Species
TGF-β	Transforming Growth Factor Beta
Th17	T Helper 17 Cells
TMAO	Trimethylamine N-Oxide
TNF-α	Tumor Necrosis Factor Alpha
VCAM-1	Vascular Cell Adhesion Molecule-1

## References

[1] Ueno M., Fujii W., Ono W., Murata H., Fujigaki Y., Shibata S. (2024). Renin inhibition and the long-term renal function in patients with hypertensive emergency: A retrospective cohort study. Am. J. Hypertens..

[2] Endo K., Hayashi K., Hara Y., Miyake A., Takano K., Horikawa T., Yoshino K., Sakai M., Kitamura K., Ito S. (2023). Impact of early initiation of renin-angiotensin blockade on renal function and clinical outcomes in patients with hypertensive emergency: A retrospective cohort study. BMC Nephrol..

[3] Angeli F., Verdecchia P., Reboldi G. (2022). Pharmacotherapy for hypertensive urgency and emergency in COVID-19 patients. Expert Opin. Pharmacother..

[4] Vilela-Martin J.F., Yugar-Toledo J.C., Rodrigues M.C., Barroso W.K.S., Carvalho L.C.B.S., González F.J.T., Amodeo C., Dias V.M.M.P., Pinto F.C.M., Martins L.F.R. (2020). Luso-Brazilian Position Statement on Hypertensive Emergencies—2020. Arq. Bras. Cardiol..

[5] Miller J.B., Hrabec D., Krishnamoorthy V., Kinni H., Brook R.D. (2024). Evaluation and management of hypertensive emergency. BMJ.

[6] Kulkarni S., Glover M., Kapil V., Abrams S.M.L., Partridge S., McCormack T., Sever P., Delles C., Wilkinson I.B. (2023). Management of hypertensive crisis: British and Irish Hypertension Society position document. J. Hum. Hypertens..

[7] Siddiqi T.J., Usman M.S., Rashid A.M., Javaid S.S., Ahmed A., Clark D., Flack J.M., Shimbo D., Choi E., Jones D.W. (2023). Clinical outcomes in hypertensive emergency: A systematic review and meta-analysis. J. Am. Heart Assoc..

[8] van den Born B.H., Lip G.Y.H., Brguljan-Hitij J., Cremer A., Segura J., Morales E., Mahfoud F., Amraoui F., Persu A., Kahan T. (2019). ESC Council on Hypertension position document on the management of hypertensive emergencies. Eur. Heart J. Cardiovasc. Pharmacother..

[9] Suneja M., Sanders M.L. (2017). Hypertensive emergency. Med. Clin. N. Am..

[10] Flanigan J.S., Vitberg D. (2006). Hypertensive emergency and severe hypertension: What to treat, who to treat, and how to treat. Med. Clin. N. Am..

[11] Rossi G.P., Rossitto G., Maifredini C., Barchitta A., Bettella A., Latella R., Ruzza L., Sabini B., Seccia T.M. (2021). Management of hypertensive emergencies: A practical approach. Blood Press..

[12] Vilela-Martin J.F., Vaz-de-Melo R.O., Kuniyoshi C.H., Abdo A.N.R., Yugar-Toledo J.C. (2011). Hypertensive crisis: Clinical-epidemiological profile. Hypertens. Res..

[13] Andrade D.O., Santos S.P.O., Pinhel M.A.S., Valente F.M., Giannini M.C., Gregório M.L., De Godoy M.F., Souza D.R.S., Vilela-Martin J.F. (2017). Effects of acute blood pressure elevation on biochemical-metabolic parameters in individuals with hypertensive crisis. Clin. Exp. Hypertens..

[14] Rossi G.P., Rossitto G., Maifredini C., Barchitta A., Bettella A., Cerruti L., Latella R., Ruzza L., Sabini B., Vigolo S. (2022). Modern management of hypertensive emergencies. High Blood Press. Cardiovasc. Prev..

[15] Haliga R.E., Cojocaru E., Sîrbu O., Hrițcu I., Alexa R.E., Haliga I.B., Șorodoc V., Coman A.E. (2025). Immunomodulatory Effects of RAAS Inhibitors: Beyond Hypertension and Heart Failure. Biomedicines.

[16] Miura S., Matsuo Y., Seumatsu Y. (2025). Renin-Angiotensin-Aldosterone System and Its Relation to Hypertension. Hypertens. Res..

[17] Zhu L., Carretero O.A., Xu J., Harding P., Ramadurai N., Gu X., Peterson E., Yang X.-P. (2015). Activation of Angiotensin II Type 2 Receptor Suppresses TNF-α-Induced ICAM-1 via NF-κB: Possible Role of ACE2. Am. J. Physiol. Heart Circ. Physiol..

[18] MacNeill E., Pade K.H. (2019). Points & pearls: Pediatric hypertension and hypertensive emergencies: Recognition and management in the emergency department. Pediatr. Emerg. Med. Pract..

[19] Koracevic G., Stojanovic M., Lovic D., Kostic T., Tomasevic M., Martinovic S.S., Zdravkovic S.C., Koracevic M., Stojanovic V. (2023). Blood pressure cut-offs to diagnose impending hypertensive emergency depend on previous hypertension-mediated organ damage and comorbid conditions. Natl. Med. J. India.

[20] Jolly H., Freel E.M., Isles C. (2023). Management of hypertensive emergencies and urgencies: Narrative review. Postgrad. Med. J..

[21] Fuchs F.D., Gus M., Gonçalves S.C., Fuchs S.C. (2023). Is it time to retire the diagnosis “hypertensive emergency”?. J. Am. Heart Assoc..

[22] Caligiuri S.P., Austria J.A., Pierce G.N. (2017). Alarming prevalence of emergency hypertension levels in the general public identified by a hypertension awareness campaign. Am. J. Hypertens..

[23] Koracevic G., Lovic D., Stojanovic M., Djordjevic D., Koracevic M. (2020). Hypertensive emergency is characterized by acute hypertension-mediated organ damage but also a life-threatening status. Hypertens. Res..

[24] Tocci G., Figliuzzi I., Presta V., Miceli F., Citoni B., Coluccia R., Musumeci M.B., Ferrucci A., Volpe M. (2018). Therapeutic approach to hypertension urgencies and emergencies during acute coronary syndrome. High Blood Press. Cardiovasc. Prev..

[25] Mathews E.P., Newton F., Sharma K. (2021). Hypertensive emergencies: A review. Am. J. Nurs..

[26] Salvetti M., Bertacchini F., Saccà G., Muiesan M.L. (2020). Hypertension urgencies and emergencies: The GEAR project. High Blood Press. Cardiovasc. Prev..

[27] Farley T.M. (2023). Hypertensive emergency: Parenteral antihypertensives and population data. Curr. Hypertens. Rep..

[28] Davis L.L. (2023). Hypertensive emergencies: Implications for nurses. Nurs. Clin. N. Am..

[29] Kotruchin P., Pratoomrat W., Mitsungnern T., Khamsai S., Imoun S. (2021). Clinical treatment outcomes of hypertensive emergency patients: Results from the hypertension registry program in Northeastern Thailand. J. Clin. Hypertens..

[30] Tocci G., Presta V., Volpe M. (2020). Hypertensive crisis management in the emergency room: Time to change?. J. Hypertens..

[31] Koracevic G., Stojanovic M., Zdravkovic M., Lovic D., Simic D., Mladenovic K. (2024). Proposal of a modified classification of hypertensive crises: Urgency, impending emergency, and emergency. Curr. Vasc. Pharmacol..

[32] Kotruchin P., Tangpaisarn T., Mitsungnern T., Sukonthasarn A., Hoshide S., Turana Y., Siddique S., Buranakitjaroen P., Van Huynh M., Chia Y.C. (2022). Hypertensive emergencies in Asia: A brief review. J. Clin. Hypertens..

[33] Kumar N., Simek S., Garg N., Vaduganathan M., Kaiksow F., Stein J.H., Fonarow G.C., Pandey A., Bhatt D.L. (2019). Thirty-day readmissions after hospitalization for hypertensive emergency. Hypertension.

[34] Cífková R., Johnson M.R., Kahan T., Brguljan J., Williams B., Coca A., Manolis A., Thomopoulos C., Borghi C., Tsioufis C. (2020). Peripartum management of hypertension: A position paper of the ESC Council on Hypertension and the European Society of Hypertension. Eur. Heart J. Cardiovasc. Pharmacother..

[35] Davis A.B., Hughes K., Pun J., Goldstein S. (2023). Hypertensive emergencies: Guidelines and best-practice recommendations. Emerg. Med. Pract..

[36] Talle M.A., Doubell A.F., Robbertse P.S., Lahri S., Herbst P.G. (2023). Clinical profile of patients with hypertensive emergency referred to a tertiary hospital in the Western Cape Province of South Africa. Curr. Hypertens. Rev..

[37] Theofilis P., Nakas N., Lamprou T., Touchantzidou K., Vordoni A., Thimis V., Smirloglou D., Kotsakis A., Kalaitzidis R.G. (2025). Hypertensive urgencies and emergencies in the cardiology emergency department: Epidemiology, patient profile, and management. J. Hum. Hypertens..

[38] Cortés Fernández M.D.S., Segura J. (2019). Urgencias hipertensivas ¿se han de tratar todas igual? [Should all hypertensive emergencies be treated in the same way?]. Hipertens. Riesgo Vasc..

[39] Brathwaite L., Reif M. (2019). Hypertensive emergencies: A review of common presentations and treatment options. Cardiol. Clin..

[40] Sauza-Sosa J.C., Zenteno-Langle R., Zamora-Medina M.D.C. (2020). Hypertensive emergency in a woman with systemic sclerosis. High Blood Press. Cardiovasc. Prev..

[41] Santamaría R., Gorostidi M. (2017). Urgencias y emergencias hipertensivas [Hypertensive urgencies and emergencies]. Hipertens. Riesgo Vasc..

[42] Khamsai S., Chootrakool A., Limpawattana P., Chindaprasirt J., Sukeepaisarnjaroen W., Chotmongkol V., Silaruks S., Senthong V., Sittichanbuncha Y., Sawunyavisuth B. (2021). Hypertensive crisis in patients with obstructive sleep apnea-induced hypertension. BMC Cardiovasc. Disord..

[43] Cucci M.D., Benken S.T. (2019). Blood pressure variability in the management of hypertensive emergency: A narrative review. J. Clin. Hypertens..

[44] Overgaauw N., Alsma J., Brink A., Hameli E., Bahmany S., Peeters L.E.J., Van Den Meiracker A.H., Schuit S.C.E., Koch B.C.P., Versmissen J. (2019). Drug nonadherence is a common but often overlooked cause of hypertensive urgency and emergency at the emergency department. J. Hypertens..

[45] Fan L., Ding L., Nie J., Wang J., Zhang M., Zhang J. (2026). Hypertensive disorders of pregnancy: A comprehensive review of pathophysiology, diagnosis, treatment, and long-term cardiovascular implications. Clin. Exp. Hypertens..

[46] Skrzypczyk P., Markiewicz M., Tutka A., Pańczyk-Tomaszewska M. (2021). Nadciśnieniowe stany pilne i nagłe u chorych pediatrycznych [Hypertensive urgencies and emergencies in pediatric patients]. Pol. Merkur. Lek..

[47] Karoui K.E. (2024). Syndrome hémolytique et urémique atypique dans sa forme associée à la grossesse, la transplantation rénale ou l’urgence hypertensive: Données récentes [Recent data on atypical hemolytic uremic syndrome associated with pregnancy, kidney transplantation or hypertensive emergency]. Nephrol. Ther..

[48] El Hussein M.T., Dolynny A. (2023). Hypertensive emergencies: Common presentations and pharmacological interventions. Crit. Care Nurs. Q..

[49] (2017). Committee Opinion No. 692 Summary: Emergent therapy for acute-onset, severe hypertension during pregnancy and the postpartum period. Obstet. Gynecol..

[50] Kotruchin P. (2023). Preliminary results of an ongoing multicentric prospective study on hypertensive emergencies and urgencies in Italy. Hypertens. Res..

[51] Paini A., Tarozzi L., Bertacchini F., Aggiusti C., Agabiti Rosei C., De Ciuceis C., Malerba P., Broggi A., Perani C., Salvetti M. (2021). Cardiovascular prognosis in patients admitted to an emergency department with hypertensive emergencies and urgencies. J. Hypertens..

[52] Paini A., Aggiusti C., Bertacchini F., Agabiti Rosei C., Maruelli G., Arnoldi C., Cappellini S., Muiesan M.L., Salvetti M. (2018). Definitions and epidemiological aspects of hypertensive urgencies and emergencies. High Blood Press. Cardiovasc. Prev..

[53] Watson K., Broscious R., Devabhakthuni S., Noel Z.R. (2018). Focused update on pharmacologic management of hypertensive emergencies. Curr. Hypertens. Rep..

[54] Janke A.T., McNaughton C.D., Brody A.M., Welch R.D., Levy P.D. (2016). Trends in the incidence of hypertensive emergencies in US emergency departments from 2006 to 2013. J. Am. Heart Assoc..

[55] Beeston D., Jepson R., Cortellini S. (2022). Evaluation of presentation, treatment and outcome in hypertensive emergency in dogs and cats: 15 cases (2003–2019). J. Small Anim. Pract..

[56] El Karoui K., Boudhabhay I., Petitprez F., Vieira-Martins P., Fakhouri F., Zuber J., Aulagnon F., Matignon M., Rondeau E., Mesnard L. (2019). Impact of hypertensive emergency and rare complement variants on the presentation and outcome of atypical hemolytic uremic syndrome. Haematologica.

[57] Saladini F., Mancusi C., Bertacchini F., Spannella F., Maloberti A., Giavarini A., Rosticci M., Bruno R.M., Pucci G., Grassi D. (2020). Diagnosis and treatment of hypertensive emergencies and urgencies among Italian emergency and intensive care departments. Results from an Italian survey: Progetto GEAR (Gestione dell’Emergenza e urgenza in ARea critica). Eur. J. Intern. Med..

[58] Krämer B.K., Krämer R.M., Benck U., Krüger B. (2019). Nonadherence in patients with hypertensive emergency or hypertensive urgency. J. Clin. Hypertens..

[59] Timmermans S.A.M.E.G., Wérion A., Damoiseaux J.G.M.C., Morelle J., Reutelingsperger C.P., van Paassen P. (2020). Diagnostic and risk factors for complement defects in hypertensive emergency and thrombotic microangiopathy. Hypertension.

[60] Roth M.A., Leyba K., Garg I., Madrid W.H., Quazi M.A., Sohail A.H., Khan R., Sultan S., Sheikh A.B. (2024). Mortality and in-patient outcomes in pheochromocytoma patients with hypertensive emergency in the United States: A propensity score matched analysis. Curr. Probl. Cardiol..

[61] Angeli F., Reboldi G., Verdecchia P. (2020). Hypertensive urgencies and emergencies: Misconceptions and pitfalls. Eur. J. Intern. Med..

[62] Vallelonga F., Cesareo M., Menon L., Leone D., Lupia E., Morello F., Totaro S., Aggiusti C., Salvetti M., Ioverno A. (2023). Hypertensive emergencies and urgencies: A preliminary report of the ongoing Italian multicentric study ERIDANO. Hypertens. Res..

[63] Taheri S., Eshgabadi A., Fatuyi M., Rahima K., Heil J. (2023). Eight-and-a-half syndrome in a patient with hypertensive emergencies. J. Hypertens..

[64] Evbayekha E., Okorare O., Ishola Y., Eugene O., Chike A., Abraham S., Aneke A.V., Green J.T., Grace A.E., Ibeson C.E. (2024). Sociodemographic predictors of hypertensive crisis in the hospitalized population in the United States. Curr. Probl. Cardiol..

[65] Amraoui F., van den Born B.H. (2021). Funduscopy in hypertensive emergencies: Detecting flames in the cotton fields. J. Clin. Hypertens..

[66] Mori H., Okamoto R., Sakaguchi S., Koji T., Dohi K. (2024). Unilateral renal artery spasm complicating hypertensive emergency in a patient with secondary aldosteronism. Eur. Heart J. Cardiovasc. Imaging.

[67] Fragoulis C., Polyzos D., Dimitriadis K., Konstantinidis D., Mavroudis A., Tsioufis P.A., Leontsinis I., Kariori M., Drogkaris S., Tatakis F. (2023). Sex-related cardiovascular prognosis in patients with hypertensive emergencies: A 12-month study. Hypertens. Res..

[68] Omar H.A., El-Bassossy H.M., Hassan N.A. (2023). Hinokitiol for hypertensive emergencies: Effects on peripheral resistance, cardiac load, baroreflex sensitivity, and electrolytes balance. Naunyn Schmiedebergs Arch. Pharmacol..

[69] Miyake A., Endo K., Hayashi K., Hirai T., Hara Y., Takano K., Horikawa T., Yoshino K., Sakai M., Kitamura K. (2024). Role of aldosterone in various target organ damage in patients with hypertensive emergency: A cross-sectional study. BMC Nephrol..

[70] Sansone R., Baaken M., Horn P., Schuler D., Westenfeld R., Amabile N., Kelm M., Heiss C. (2018). Release of endothelial microparticles in patients with arterial hypertension, hypertensive emergencies and catheter-related injury. Atherosclerosis.

[71] Parrelli G.A., Musey P.I., Kuhn D. (2026). Hypertensive emergency department patients frequently have no existing hypertension diagnosis or antihypertensive medications. J. Hypertens..

[72] Bress A.P., Anderson T.S., Flack J.M., Ghazi L., Hall M.E., Laffer C.L., Still C.H., Taler S.J., Zachrison K.S., Chang T.I. (2024). The Management of Elevated Blood Pressure in the Acute Care Setting: A Scientific Statement From the American Heart Association. Hypertension.

[73] Li W., Chen D., Liu S., Wang X., Chen X., Chen J., Ma J., Song F., Li H., Yan S. (2021). The Rates and the Determinants of Hypertension According to the 2017 Definition of Hypertension by ACC/AHA and 2014 Evidence-Based Guidelines Among Population Aged ≥40 Years Old. Glob. Heart.

[74] Chioncel O., Mebazaa A., Harjola V.P., Coats A.J., Piepoli M.F., Crespo-Leiro M.G., Laroche C., Seferovic P.M., Anker S.D., Ferrari R. (2017). Clinical Phenotypes and Outcome of Patients Hospitalized for Acute Heart Failure: The ESC Heart Failure Long-Term Registry. Eur. J. Heart Fail..

[75] Benenson I., Waldron F.A., Holly C. (2023). A systematic review and meta-analysis of the clinical and epidemiological characteristics of patients with hypertensive emergencies: Implication for risk stratification. High Blood Press. Cardiovasc. Prev..

[76] Fragoulis C., Dimitriadis K., Siafi E., Iliakis P., Kasiakogias A., Kalos T., Leontsinis I., Andrikou I., Konstantinidis D., Nihoyannopoulos P. (2022). Profile and management of hypertensive urgencies and emergencies in the emergency cardiology department of a tertiary hospital: A 12-month registry. Eur. J. Prev. Cardiol..

[77] Shah M., Patil S., Patel B., Arora S., Patel N., Garg L., Agrawal S., Jacobs L., Steigerwalt S.P., Martinez M.W. (2017). Trends in hospitalization for hypertensive emergency, and relationship of end-organ damage with in-hospital mortality. Am. J. Hypertens..

[78] Savigny E., Gobin N., Amyai N. (2022). Hypertension artérielle à l’hôpital: Faut-il la traiter? [High blood pressure in hospital: Should it be treated?]. Rev. Med. Suisse.

[79] Pierin A.M.G., Flórido C.F., Santos J.D. (2019). Hypertensive crisis: Clinical characteristics of patients with hypertensive urgency, emergency and pseudocrisis at a public emergency department. Einstein.

[80] Robles N.R., Fici F., Grassi G. (2024). Management of hypertensive urgencies: A new opportunity for unattended blood pressure measurement. J. Hum. Hypertens..

[81] Maghraoui H., Zaouche K., Yahya Y., Mghirbi A., Ben Hamida G., Majed K. (2019). Care of hypertensive urgencies and emergencies in the emergency department. Tunis. Med..

[82] Nacanabo W.M., Seghda T.A.A., Dah D.C., Sawadogo W.L.F., Sawadogo I., Dimzouré M.S., Loya M., Thiombiano L.P., Samadoulougou A.K. (2024). Urgences hypertensives au Centre hospitalier universitaire de Bogodogo, Ouagadougou (Burkina Faso) [Hypertensive emergencies at Bogodogo University Hospital, Ouagadougou (Burkina Faso)]. Med. Trop. Sante Int..

[83] Koracevic G., Lovic D., Zdravkovic M., Stojanovic M., Djordjevic D. (2020). How should we treat very high blood pressure until we distinguish between hypertensive emergency and urgency?. Hypertens. Res..

[84] Nijskens C.M., Veldkamp S.R., Van Der Werf D.J., Boonstra A.H., Ten Wolde M. (2021). Funduscopy: Yes or no? Hypertensive emergencies and retinopathy in the emergency care setting: A retrospective cohort study. J. Clin. Hypertens..

[85] Tran Q.K., Gelmann D., Zahid M., Palmer J., Hollis G., Engelbrecht-Wiggans E., Alam Z., Matta A.E., Hart E., Haase D.J. (2023). Arterial monitoring in hypertensive emergencies: Significance for the Critical Care Resuscitation Unit. West. J. Emerg. Med..

[86] Waldron F.A., Benenson I., Jones-Dillon S.A., Zinzuwadia S.N., Adeboye A.M., Eris E., Mbadugha N.E., Vicente N., Over A. (2019). Prevalence and risk factors for hypertensive crisis in a predominantly African American inner-city community. Blood Press..

[87] Giani V., Valobra T., Capsoni N., Galasso M., De Censi L., Ferretti C., Sultana A., Giacalone A., Garofani I., Bombelli M. (2024). Neurological hypertensive emergencies: Correlation of blood pressure values with in-hospital outcomes in ischemic stroke. Eur. J. Intern. Med..

[88] Sims T.D., Shubert L., Denning S., Shay L., Green C., Davidson C. (2025). Impact of a maternal early warning system and severe hypertension safety bundle on timely treatment of hypertensive emergencies: A quality improvement and health equity initiative. BJOG.

[89] Grogan L., Peterson E., Flatley M., Domeyer-Klenske A. (2025). Impact of patient safety bundle and team-based training on obstetric hypertensive emergencies. Am. J. Perinatol..

[90] Vilela-Martin J.F., Vaz-de-Melo R.O., Cosenso-Martin L.N., Kuniyoshi C.H., Yugar-Toledo J.C., Pinhel M.A., de Souza G.F., Souza D.R., Pimenta E., Moreno H. (2013). Renin–Angiotensin System Blockage Associates with Insertion/Deletion Polymorphism of Angiotensin-Converting Enzyme in Patients with Hypertensive Emergency. DNA Cell Biol..

